# Active colloids under geometrical constraints in viscoelastic media

**DOI:** 10.1140/epje/s10189-021-00033-w

**Published:** 2021-03-11

**Authors:** N Narinder, Wei-jing Zhu, Clemens Bechinger

**Affiliations:** 1 Fachbereich Physik, Universität Konstanz, Konstanz, Germany; 2 School of Physics and Telecommunication Engineering, South China Normal University, Guangzhou, China; 3 School of Photoelectric Engineering, Guangdong Polytechnic Normal University, Guangzhou, 510665 China

## Abstract

**Abstract:**

We study the behavior of active particles (APs) moving in a viscoelastic fluid in the presence of geometrical confinements. Upon approaching a flat wall, we find that APs slow down due to compression of the enclosed viscoelastic fluid. In addition, they receive a viscoelastic torque leading to sudden orientational changes and departure from walls. Based on these observations, we develop a numerical model which can also be applied to other geometries and yields good agreement with experimental data. Our results demonstrate, that APs are able to move through complex geometrical structures more effectively when suspended in a viscoelastic compared to a Newtonian fluid.

**Graphic Abstract:**

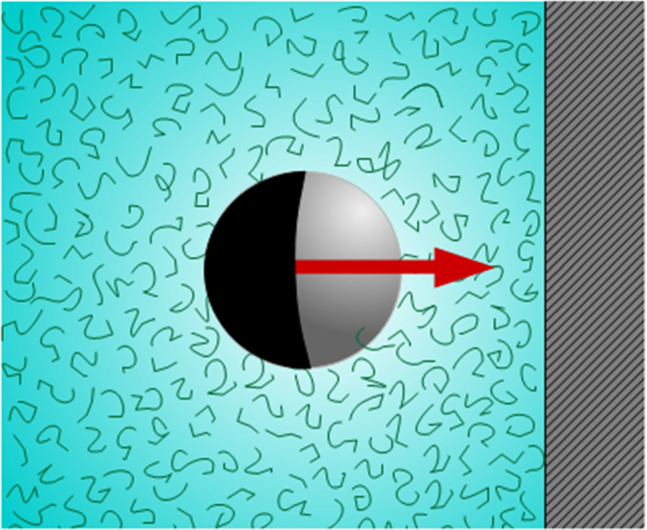

## Introduction

Active particles (APs) currently receive great attention because they display striking similarities with living active matter [1–6]. Being able to respond to, e.g., chemical [7] and optical intensity gradients [8], flow [9] and gravitational fields [10], they share many properties with microorganisms. As a result of their drastically reduced complexity and shape rigidity, APs have been recognized as versatile model systems to explore in detail the swimming motion of microorganisms under well controlled conditions [11]. Compared to the majority of experimental and theoretical studies which have investigated the behavior of APs in purely viscous media, i.e., Newtonian fluids [12], however, the motion of APs in viscoelastic media is only poorly understood even though they provide the natural environment of many motile bacteria and cells [13–15]. Recent experiments revealed a number of striking properties of APs in viscoelastic fluids including a strongly enhanced rotational diffusion coefficient [35, 36] and even trajectories with a persistent circular shape [16]. Notably, these experiments have been performed at rather low propulsion velocities, i.e., far beyond those leading to nonlinear rheological properties such as shear thinning [17–20]. Apart from the properties of the swimming medium which has a large impact on the AP motion, this also applies to the presence of additional topographical features. Already in case of viscous fluids, the presence of walls, channels and even more complex structures has been reported which lead to drastic changes in the AP motion [21–34].

**Fig. 1:**
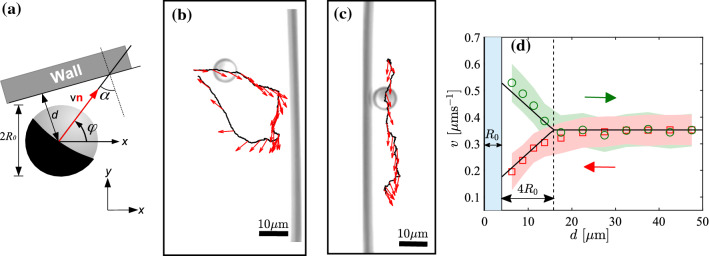
**a** Sketch of a Janus particle (diameter $2R_0$) near a flat wall. **b** Typical AP trajectory when approaching a wall with almost parallel ($\alpha \approx 0$ rad) and **c** perpendicular ($\alpha \approx \pi /2$ rad) orientation normal to the wall. The actual AP orientation is denoted by red arrows which are shown for time intervals of 25 s each. **d** Particle velocity as a function of its distance *d* to the wall during approach ($\square$) and departure ($\bigcirc$). The shaded areas represent the variations observed during 25 trajectories. The solid lines correspond to the *v*(*d*) dependence used in our numerical simulations

Here, we study the motion of APs within viscoelastic media and additionally confined to walls, circular pores and snake-like channels. We find pronounced differences compared to their behavior in Newtonian fluids regarding their interaction with walls but also their particle distribution. Based on our experimental observations, we present a phenomenological Langevin description which is able to quantitatively reproduce our experimental findings and can be extended to more complex confinement geometries. In particular, our results demonstrate that viscoelastic fluids can strongly increase the ability of APs to explore complex geometrical structures within a given time. This may be useful for their future application as micro-agents and shuttles in fluid environments.

## Experimental details

The viscoelastic swimming medium was comprised of polyacrylamide (0.05 wt%) dissolved in water. In addition, we added propylene glycol $n-$propyl ether (PnP) with mass ratio 2:3 to this solvent which renders the entire fluid to become critical at a lower demixing temperature $T_c=304.6$ K [35]. Using a bath thermostat, the temperature of the mixture was kept at $T=298$ K during our experiments. The rheological properties [39] of the system have been probed using passive microrheology. The fluid’s zero shear viscosity $\eta _0$ and the stress relaxation time $\tau$ were determined to 0.210 Pa s and 17.27 s, respectively. As APs, we used silica spheres (radius, $R_0=3.95$ $\upmu$m) which are half-coated with a 30-nm carbon layer and which were added to the critical fluid and confined in a cell of height $h\approx 4R_0$. Due to gravity, the particles sediment toward the bottom plate, where their translational and orientational motion is confined to two dimensions (2D) [37]. To make the particles active, the entire sample is homogeneously illuminated with a green laser ($\lambda =532$ nm). Due to the selective absorption of the laser light at the carbon caps, the surrounding fluid becomes inhomogeneously heated which leads to a local demixing of the fluid and thus to particle propulsion whose velocity *v* can be controlled by the light intensity [8, 37, 38]. For the intensities considered here, the particles propel opposite to the carbon cap and their velocities are varied from $0.1~\upmu$ms$^{-1}$ to $0.4~\upmu$ms$^{-1}$, which corresponds to a Péclet number (Pe$=v_0R_0/D$) and Weissenberg number (Wi$=v_0\tau /2R_0$) ranging from $\approx 150-700$ and $\approx 0.2-0.85$, respectively. Topographical patterns on the glass substrate are fabricated by photolithography using the photoresist SU-8. Particle positions (*x*, *y*) and their in-plane orientation $\mathbf{n} =(\mathrm {cos}\varphi ,\mathrm {sin}\varphi )$ were tracked using a standard image processing algorithm [40].

## AP dynamics near walls and in pores

In order to characterize the motion of APs swimming near walls, in the following we describe their position and orientation relative to the wall as shown in Fig. 1a. The shortest AP distance and orientation relative to the wall are denoted by *d* and $\alpha$, respectively. When observing the motion of APs near walls in our experiments, we generally find that their velocity *v* decreases substantially when moving toward the wall (Fig. 1b). When approaching the wall, we find a drop in velocity by $v \approx v_0/2$, with $v_0$ the AP velocity in the bulk, i.e., far away from the wall. After the AP has reached its closest distance to the wall, it experiences a sudden reorientation which leads to departure from the wall. While leaving the wall, its velocity becomes enhanced up to ($v\approx 3v_0/2$). The entire approaching/departure dynamics also depends on the initial orientation $\alpha$ of the AP relative the wall. This is seen in Fig.1c where we show the situation for ($\alpha \approx 0$ rad) where the AP mainly slides along to the wall. The AP–wall interaction observed here is very different compared to what is observed in case of a viscous fluid [21, 41]. In the latter case, the velocity of APs remains unchanged while approaching the wall. This suggests that the reduction in *v* in the former case essentially stems from the effective compression of the enclosed viscoelastic fluid between AP and the wall. When the AP approaches the wall, it typically remains near it on the order of its rotational diffusion time $\tau _\varphi =1/D_\varphi$. For the viscosity and AP diameter in our experiments, this would correspond to $\sim 22$ hours. In contrast to that, in our experiments the APs stay only for about $\sim 500$ s near the wall.

The above mentioned observations are qualitatively explained by the accumulation and relaxation of mechanical stress within the viscoelastic fluid between the AP and the wall [41, 42]. An AP moving toward the wall induces a strain in the enclosed fluid’s polymer network. Moving against the strained fluid dramatically hinders its motion leading to the observed decrease in velocity. Because of spatial fluctuations, this strain field leads not only to a change in the translational particle velocity but also couples to its orientation dynamics which gives rise to a viscoelastic torque. This is eventually responsible for the observed fast AP reorientation near the wall. The reorientation away from the wall leads to a sudden release of the accumulated stress and thus strongly increases the AP’s departing velocity. Notably, the observed velocity variations near the wall are rather independent of the angle of incidence angle $\alpha$. The results averaged over 25 trajectories for particles approaching (squares) and departing (circles) toward and from the wall are shown in Fig. 1d. As seen, the wall-induced variation of the velocity is, for the chosen value of $v_0=0.35~\upmu$ms$^{-1}$, observed for particle-wall distances closer than $4R_0$. This distance is attributed to the interaction range between an AP and a wall mediated by the viscoelastic fluid.

**Fig. 2:**
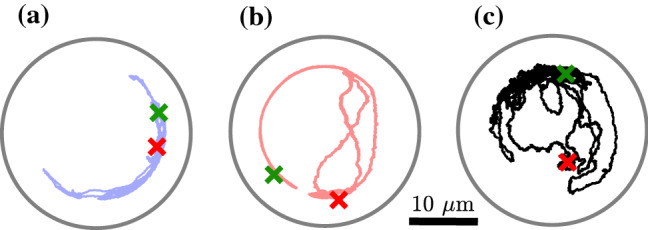
**a**–**c** Representative trajectories of an AP moving inside a circular pore with radius $R_\mathrm {p}=15\upmu$m in viscoelastic fluid (a) $v_0=0.2~\upmu$ms$^{-1}$, (b) $v_0=0.3~\upmu$ms$^{-1}$ and, (c) $v_0=0.4~\upmu$ms$^{-1}$. The symbols ($\times$) mark the starting (green) and final (red) AP position after 3600 s

**Fig. 3:**
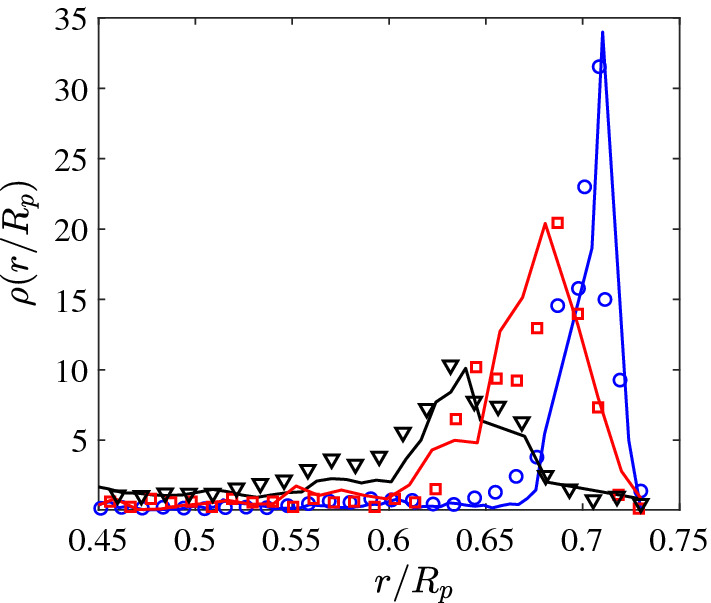
The probability density function of the radial position $\rho (r/R_p)$ of APs having different velocities $v_0=0.2~\upmu$ms$^{-1}$ ($\bigcirc$), $v_0=0.3~\upmu$ms$^{-1}$ ($\square$) and, $v_0=0.4~\upmu$ms$^{-1}$ ($\bigtriangledown$). The solid lines are corresponding $\rho (r/R_p)$ obtained from numerical simulations

In addition to flat walls, we also have studied the motion of APs inside circular pores with radius $R_\mathrm {p}=15~\upmu$m. Under such conditions, AP–wall encounters are more frequent and viscoelastic interactions with the walls become more pronounced. Figure 2a–c shows typical particle trajectories (3600 s) of the same AP but for different propulsion velocities $v_0$. With increasing $v_0$, the AP is found more often near the pore center (i.e., away from its walls) which leads to a systematic shift of the radial probability distribution $\rho (r/R_p)$ toward smaller distances with increasing $v_0$ (Fig. 3). We interpret this behavior to result from increasing reorientation events upon AP–wall collisions as discussed above.

## Phenomenological modelling and numerical results

The experimental observations presented in Sect. 3 highlight two key features of the AP dynamics in a viscoelastic fluid close to walls: First, the presence of walls strongly modifies their velocity depending on its distance *d* relative to wall. Second, an AP close to the wall also a experiences viscoelastic torque which strongly depends on its distance *d* and angle $\alpha$ relative to wall.

Motivated by these findings, in the following we present a phenomenological description of the 2D particle motion $\mathbf {r(t)}=(x(t),y(t))$ via the following Langevin equations

1 $$\begin{aligned} \frac{\mathrm {d}\mathbf {r}}{\mathrm {d} t}=\mathbf {v}(d,\alpha )+\sqrt{2D}\varvec{\xi }_r \end{aligned}$$

with *D* the translational diffusion coefficient and $\varvec{\xi }_r$ is the translational Gaussian noise which mimics the thermal fluctuations and is characterized by $\left\langle \varvec{\xi }_r \right\rangle =0$ and $\left\langle \xi _r^i(t)\xi _r^j(t') \right\rangle =\delta _{ij}\delta (t-t')$. The change of the AP velocity on its wall distance *v*(*d*) during approach and departure from the wall is obtained from a linear fit to experimental data (solid lines in Fig. 1d) and is given as: For approach

2 $$\begin{aligned} v(d)= \frac{v_0}{6}\left( \frac{d}{R_0}+2\right) . \end{aligned}$$

For departure

3 $$\begin{aligned} v(d)=-\frac{v_0}{6}\left( \frac{d}{R_0}-10\right) . \end{aligned}$$

In accordance with our experimental observations, the velocity was set to $v_0$ beyond the viscoelastic interaction range $4R_0$.

In addition to the translational AP motion, its angular dynamics is modelled via

4 $$\begin{aligned} \frac{\mathrm {d}\varphi }{\mathrm {d} t}=\omega (d,\alpha )+\sqrt{2D_\varphi (v_0)}{\xi }_\varphi . \end{aligned}$$

**Fig. 4:**
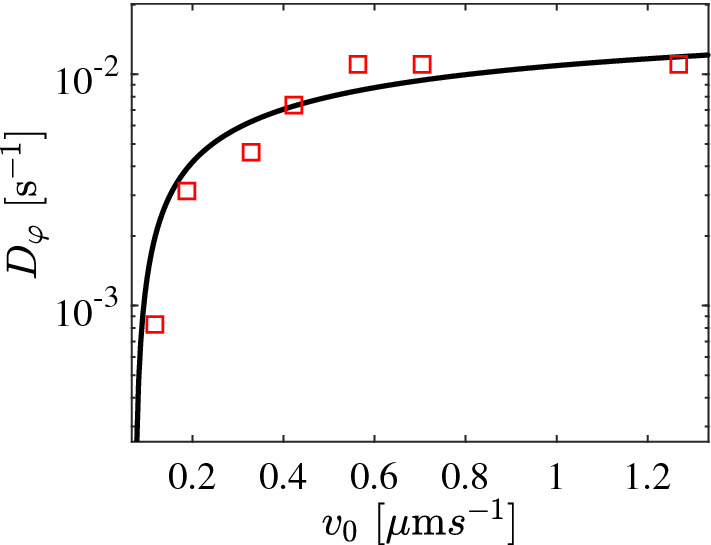
Rotational diffusion coefficient $D_\varphi$ of an AP ($R_0=3.95~\upmu$m) as a function of AP velocity $v_0$ in a viscoelastic fluid. The symbols are measured experimentally from the long-time mean squared displacements, and the solid line is the fit according to $D_\varphi (v_0)=0.5\times v_0^{0.008}-0.492$

Here, $$D_\varphi (v_0)$$ is the rotational diffusion coefficient of the AP. Contrary to Newtonian fluids where $$D_\varphi $$ is independent of $$v_0$$, it has been demonstrated that this is no longer the case in viscoelastic fluids [35, 36]. Figure 4 shows the measured $$D_\varphi $$ of an AP vs $$v_0$$ taken from the measured long-time behavior of the mean squared displacement. For an AP, the data are well described by a phenomenological expression $$D_\varphi (v_0)=0.5\times v_0^{0.008}-0.492$$. This relationship has been used in our numerical simulations. $$\xi _\varphi $$ denotes the angular noise with $$\left\langle \xi _\varphi \right\rangle = 0$$ and $$\left\langle \xi _\varphi (t)\xi _\varphi (t') \right\rangle =\delta (t-t')$$. The experimentally observed viscoelastic torques are expressed by the term $$\omega (d,\alpha )$$ which is not known *a priori*. For simplicity, we have assumed $$\omega (d,\alpha )$$ to be a gaussian function centered around $$\alpha =0$$ rad. Furthermore, $$\omega (d,\alpha )$$ is set to zero for $$ d>1.8 R_0$$. This is motivated by our observations, that sudden changes in the direction of APs upon approaching the wall only occur below this value (Fig. 1b). In principle, the translational diffusion coefficient *D* also depends on the velocity [35]. This velocity dependence, however, is much smaller compared to $$D_\varphi $$ and has already reached its saturation regime for the propulsion velocities considered here. Therefore, in Eq. 1*D* was kept constant at the saturation value $$D = 2.63 \times 10^{-3} \upmu $$m$$^2$$/s.

**Fig. 5:**
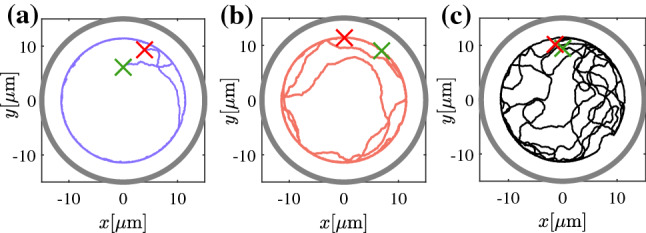
Numerical trajectories of APs moving inside circular confinements of radius $15~\upmu$m at **a** $v_0=0.2~\upmu$ms$^{-1}$, **b** $v_0=0.3~\upmu$ms$^{-1}$, **c** $v_0=0.4~\mu$ms$^{-1}$ over 3600 s. The green and red crosses mark the initial and final position of the particle respectively

**Fig. 6:**
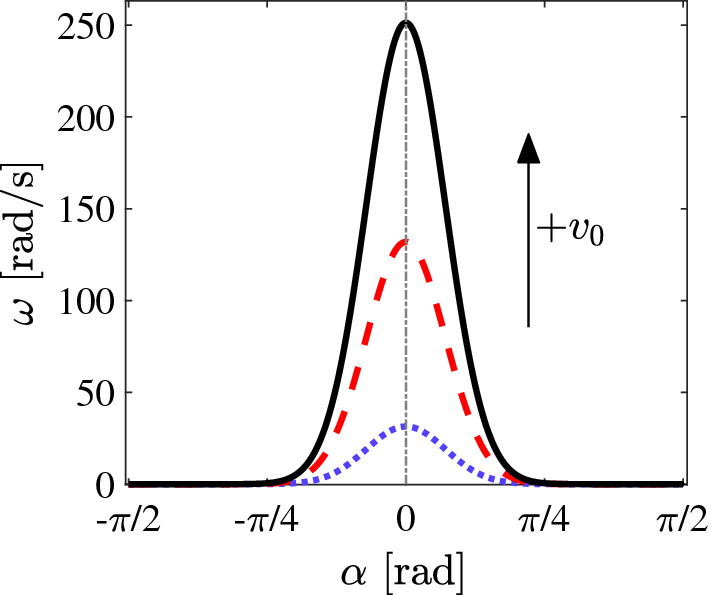
Angular rotation $\omega$ for $d\le 1.8R_0$ as a function of the AP angle with respect to wall, starting from below for $v_0=0.2~\upmu$ms$^{-1}$ (dotted line), $v_0=0.3~\upmu$ms$^{-1}$ (dashed line) and $v_0=0.4~\upmu$ms$^{-1}$ (solid line), respectively, imposed numerically on the active particle for obtaining qualitative agreement with experiments

Using these Langevin equations, we have computed AP trajectories within circular pores with identical dimensions as those in the experiments shown in Fig. 2. The results for different $v_0$ are shown in Fig. 5a–c. As adjustable parameters, we have varied the height and width of the gaussian distributions of $\omega (\alpha )$ such, that best qualitative agreement with our experimental trajectories is obtained (Fig. 6). To appreciate the agreement between experimental data and our phenomenological model, we have also computed the numerically obtained radial distribution functions which compare very well with those measured in our experiments ( solid lines in Fig. 3).

## Application of model to the dynamics in a snake-like channel

**Fig. 7:**
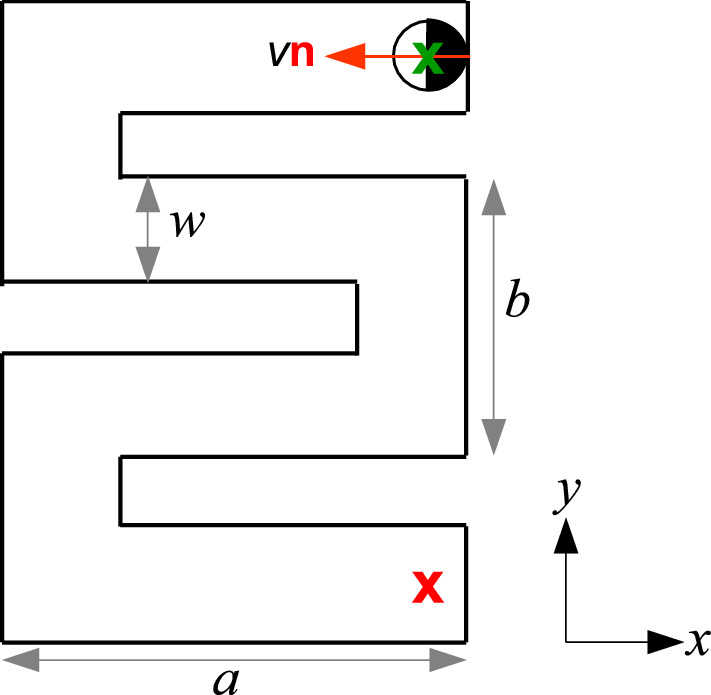
Sketch of the snake-like channel with the initial position and the orientation of the particle. The crosses mark the initial (green) and final (red) positions of the particle. The dimensions *a*, *b* and the width of cross section *w* of the channel are $80~\upmu$m, $50~\upmu$m and, $20~\upmu$m, respectively

**Fig. 8:**
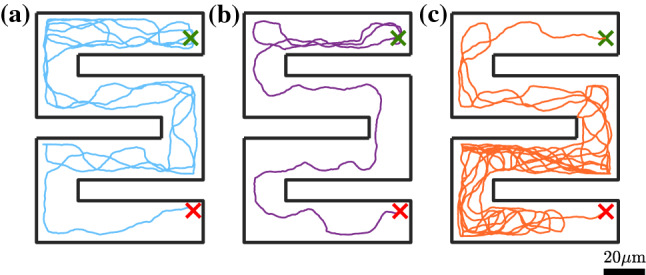
Numerical trajectory of active particle from initial position (green cross) and orientation ($\varphi =\pi$ rad) to the final position which is the other end of the channel (red cross) moving at different velocities **a** $v=0.225~\upmu$ms$^{-1}$, **b** $v=0.325~\upmu$ms$^{-1}$ and **c** $v=0.4~\upmu$ms$^{-1}$

**Fig. 9:**
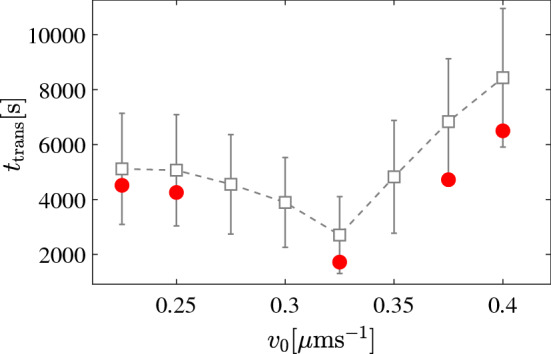
Average time it takes for an AP to cross the structure (from the upper right to the lower right corner) having an initial orientation ($\varphi =\pi$ rad) as a function of $v_0$. The open and filled symbols correspond to numerical and experimental data

**Fig. 10:**
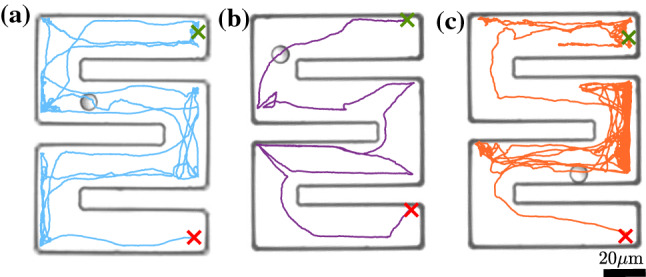
Exemplary experimental trajectory of active particle from initial position (green cross) and orientation ($\varphi =\pi$ rad) to the final position (red cross) at various propulsion velocities **a** $v=0.225~\upmu$ms$^{-1}$, **b** $v=0.325~\upmu$ms$^{-1}$ and **c** $v=0.4~\upmu$ms$^{-1}$

We now apply our model to another more complex geometry. In the following, we consider a snake-like channel with dimensions $a=80~\upmu$m, $b=50~\upmu$m and, $w=20~\upmu$m (Fig. 7). For this, we computed particle trajectories with fixed initial position (upper right corner) and orientation and for different propulsion velocities [see Fig. 7]. Figure 8a–c shows typical trajectories with the identical parameters used in the previous simulations [Eqs. 1–4]. Compared to the motion in a Newtonian fluid where APs tend to remain near walls and get easily stuck in corners, here the particles seem to explore the entire structure rather rapidly. This is largely promoted by the frequent reflections from the walls due to the viscoelastic torques. To quantify the efficiency an AP explores the entire structure, we have calculated the mean transition time $t_{\text {trans}}$ from the initial position (green cross) to the lower right corner (red cross) averaged over 20 runs each (open symbols in Fig. 9). As expected $t_{\text {trans}}$ decreases with increasing propulsion velocity but then strongly increases above $v_0 = 0.325~\upmu$ms$^{-1}$. To understand this—at first glance—strange behavior, we recall that both the angular rotation and the rotational diffusion increase with $v_0$ (Figs. 4 and 6). This eventually leads to back and forth reflections between two parallel walls which can temporarily trap the AP (see Fig. 8c). In addition to numerical simulations, we also performed experiments with the identical snake-like geometry. Typical trajectories which are shown in Fig. 10a–c compare well to those in Fig. 8a–c. In particular, the back and forth reflections at higher propulsion velocities are nicely reproduced by our data. When comparing $t_{\text {trans}}$ to the numerical data, within error bars we find good agreement. Even though experimental and numerical data agree within the error bars, the experimental transition times are systematically below those obtained from the simulations. This difference is caused by the back and forth AP reflections between regions of the snake-like channels where the AP is confined between two closely spaced parallel walls. When comparing Figs. 8 and 10, one finds (in particular at large AP velocities) that such reflections lead to an effective AP trapping at those sites which is more pronounced in the simulations compared to the experiments. We believe this is caused by non-additive effects of the viscoelastic interaction of APs in presence of two nearby walls which is not considered in our simulations. The observed non-monotonic behavior in $t_{\text {trans}}$ should be absent in viscous fluids, since the orientational particle dynamics under such conditions is independent of $v_0$. Accordingly, we expect that $t_{\text {trans}}$ does not exhibit a minimum but rather monotonically decays with $v_0$. Once the persistence length is larger than the typical geometrical structure, we expect $t_{\text {trans}}$ to saturate.

## Conclusions

In conclusion, we have investigated the dynamics of active particles in a viscoelastic fluid close to a single flat wall, a cylindrical pore and a snake-like channel. We show that their behavior is governed by pronounced changes in their velocity upon approaching and departing from walls. During AP reflection, they receive a viscoelastic torque which dominates their orientational dynamics. These behaviors are induced by the accumulated and released stresses in the enclosed viscoelastic medium. Given the fact that the identical parameters in the model are able to reproduce the AP behavior in circular pores and snake-like channels, this suggests that the relevant effects for AP motion under such conditions have been captured by our phenomenological numerical model. Therefore, we expect that similar agreement is also found for other structures.
